# Towards Improving the Ethics of Ecological Research

**DOI:** 10.1007/s11948-014-9558-4

**Published:** 2014-06-06

**Authors:** G. K. D. Crozier, Albrecht I. Schulte-Hostedde

**Affiliations:** 1 Canada Research Chair in Environment, Culture and Values, Laurentian University, Sudbury, ON Canada; 2 Canada Research Chair in Applied Evolutionary Ecology, Laurentian University, Sudbury, ON Canada

**Keywords:** Ecology, Bioethics, Philosophy of biology, Professional ethics, Animal research ethics, Decision theory, Ethics code, Ethics education

## Abstract

We argue that the ecological research community should develop a plan for improving the ethical consistency and moral robustness of the field. We propose a particular ethics strategy—specifically, an ongoing process of collective ethical reflection that the community of ecological researchers, with the cooperation of applied ethicists and philosophers of biology, can use to address the needs we identify. We suggest a particular set of conceptual (in the form of six core values—freedom, fairness, well being, replacement, reduction, and refinement) and analytic (in the forms of decision theoretic software, 1000Minds) tools that, we argue, collectively have the resources to provide an empirically grounded and conceptually complete foundation for an ethics strategy for ecological research. We illustrate our argument with information gathered from a survey of ecologists conducted at the 2013 meeting of the Canadian Society of Ecology and Evolution.

## An Ethics Strategy—Why?

### Introduction

‘Ecological research’ is the branch of biological research focused on the relationships among organisms, their groups, and their environments. In the academic and popular literature, it has been recognized that choices made by ecological researchers when designing and managing their field experiments have strong and varied ethical implications, and that some systematic method for reflecting on the ethical dimensions of experimental design in ecological field studies is needed. Decisions made regarding what to study and how to do so often impact the studied ecosystems, fellow researchers, local communities, policy decisions, and even the progress of Ecology. Even purely observational studies that are designed to minimize any disruption to the organisms under observation frequently affect their subjects or local human communities (Strier 2010).

Consider the following two examples of ethical challenges faced by Canadian ecological researchers.

#### Example I

A research team has an extraordinarily successful long-term study of a population of bighorn sheep (*Ovis canadensis*) on Ram Mountain, an isolated outcrop adjacent to the Canadian Rocky Mountains (Festa-Bianchet and Jorgenson 2002; Festa-Bianchet et al. 2014). The population contains marked individuals for which the research team has incredibly detailed data on phenotype, pedigree, and life-history. Many graduate students, post-doctoral fellows, and senior scientists have studied this population, and this research has lead to numerous important publications. Recently, however, a cougar (*Puma concolor*) that has learned to specialize on these sheep is slowly but surely eating all of them. This is a study of a natural population, which includes predation, but this cougar is drastically reducing the sample size of the study. Since it is legal to hunt cougars in the region where this study is taking place, one option is to try to kill the predator; however, even if a cougar were successfully hunted, this would not ensure that it was the correct one. What ought to be done?

#### Example II

An ecological researcher conducted a field experiment that involved translocating milkweed (*Asclepias syriaca*) specimens to a range of sites across a large geographical area (Woods et al. 2012). After studying how the plants adapted to the diverse conditions provided by these ecosystems, he opted to destroy the milkweed gardens by treating them with herbicide. He reasoned that “there is phylogeographic structure in the population genetics of the plants, and this structure might be of interest to an ecologist in the future” (Stephen Heard, personal communication). However, no standard accepted protocol has been established for field studies involving the transplantation of organisms; although ecological researchers are highly attuned to the dangers posed by ‘species invasion,’ which can occur when non-native organisms are introduced into an ecosystem where there are no native conspecifics, less attention is paid to the potential detrimental impacts of artificially enhancing gene flow within the native range of a species. This should be of additional concern for ecologists that conduct transplant experiments because, by artificially promoting gene flow within a species’ range via transplantation, the result can be disruption of local adaption, and thus reduced Darwinian fitness. Ultimately, this has ecological and evolutionary consequences for the focal population, as well as affecting any future research. Should this concern be more widespread? What might this reasoning entail in cases where the translocated species are not plants, but animals, where destroying the test subjects would be more contentious?

At present, ethically salient decisions in ecological research are based on a patchwork of disconnected and collectively incomplete ethical guidelines within the literature and practices of the ecology community. Exacerbating the inadequacy of ethical discussion and guidance regarding ethical research design is the rarity of any ethics training received by ecology graduate students. Furthermore, as one ecologist put it: “People studying biology are particularly illiterate in ethics since students with ethical concerns about use of animals are steered to other non-scientific fields. Such concerns are viewed as invalid. In fact, it is often the case that ‘we’ (ecologists) are ill informed. Discourses of ethical concerns for animals remain largely taboo.”^1^ Accordingly, those entering the ecology research community might not share a common ethical vocabulary (more sophisticated than the colloquialisms of ‘folk’ ethics) necessary for the efficient communication of the ethical implications of their research decisions; and they might not possess the corresponding set of conceptual tools to help them efficiently identify and address ethical issues arising in field study design.

It is incumbent upon the ecological research community to develop a strategy or program for improving the ethical consistency and moral robustness of the field. To this end, we propose a particular approach—specifically, an ongoing process of collective ethical reflection that the community of ecological researchers, with the cooperation of applied ethicists and philosophers of biology, can use to investigate and improve the ethical dimensions of their field research. At the center of this process are six core values (justice, freedom, well-being, reduction, refinement, and replacement) and a decision theoretic tool (an online, multi-criteria decision analysis software, 1000Minds) that, together, can ensure that recommendations regarding ethical design of field studies are empirically grounded in the practical experiences and concerns of working ecologists. It is our expectation that this ongoing process can assist the ecological research community to clarify ethically salient desiderata of research design such that: (a) morally significant decisions can be made under a variety of conditions, including conditions of uncertainty; and (b) new cases can to be dealt with in a way that is either consistent with older cases, or where inconsistencies are justifiable as exceptional or precedent-setting.

### Literature and Context

In light of the escalating urgency of pollution, climate change, and other destructive anthropogenic phenomena, ecological researchers are under increasing pressure to innovate and refine their methods (Minteer and Collins 2008), to modify the way they communicate their results with policy makers (Baskerville 1997), and to shift the focus of their research (Goldenberg 2012; Shen 2013). Furthermore, decisions regarding the manner and timing by which the results of ecological research are communicated to decision makers can contribute significantly to the uncertainty levels under which key decisions are made (Baskerville 1997; Wiens 1997; Schwartz et al. 2012).^2^

Nevertheless, relatively little explicit attention is given in the academic literature to decisions about experimental design that ecological researchers make for ethical reasons. Although several ecological (Schrader-Frechette and McCoy 1993; Franz 2001; Falkenmark and Folke 2002; Cairns 2003; Wallington and Moore 2005; Minteer and Collins 2005a, 2005b, 2008, 2010; Parris et al. 2010; Schwartz et al. 2012) and philosophical (Cooper 2003; Norton and Noonan 2007; MacLaurin and Sterelney 2008; Odenbaugh 2003, 2008; Colyvan et al. 2009) papers note concern with the ethical challenges that persist within ecological research and make varied recommendations, little measurable progress has been made toward addressing this shortcoming.

While environmental ethics,^3^ conservation ethics, and animal research ethics have developed extensive literatures, relatively little has been published on the ethics of ecological research and field studies. Similarly, discussion of the ethical criteria that have been applied by working ecologists during the design of their field studies are not discussed in the ‘methods’ sections of their research publications, nor do they receive attention in the vast majority of conference presentations.

It is generally left to the judgment of the individual biologist to decide when disturbance due to field practices is justifiable. The paucity of discussion of these issues in the literature makes it difficult to assess how individual scientists make these decisions, or how the sum of these decisions affects the organisms that we study and the science that we do.

(Farnsworth and Rosovsky 1993)

Furthermore, ecological journals typically do not scrutinize the ethical dimensions of the research they publish, aside from ensuring that externally imposed regulations have been met (Marsh and Eros 1999; Marsh and Kenchington 2004).

Farnsworth and Rosovsky (1993) identified three reasons why there is negligible discussion in ecological scientific publications on ethical considerations underlying decisions about experimental design. First, ecologists are worried that widening the discussion of design methods to explicitly include ethical considerations will invite others to police and otherwise constrain future research efforts. Second, there is a tacit assumption among ecologists (and indeed among researchers of all types) that the long-term benefits of ecological research outweigh short-term costs—an assumption that could be true, but could just as easily be the result of bias. Third, it can be very challenging to perceive or quantify the impact of various ecological field studies, thus confounding efforts to reliably evaluate the impact—and the ethically salient consequences—of particular research activities.^4^

One notable exception arose in 1996 when marine ecologists sought permission to perform a large-scale experiment on coral colonies in the Great Barrier Reef to assess the effects of line fishing—after a highly publicized legislative battle, the research was deemed too destructive to the marine life and was not permitted (Marsh and Kenchington 2004). One result of this Line Fishing case was the development of ethical guidelines in Australia designed to minimize the impact of field experiments on high-risk ecosystems (ASTEC 1998).

The Line Fishing case can serve to illustrate two relevant trends. First, the existing ethical guidelines that impact ecologists (some developed internally to the profession, and some imposed externally) do not individually or collectively provide comprehensive guidance on the ethics of ecological research; rather, each focuses on some aspect such as minimizing harm to animals, protecting high-risk sites, or protecting endangered species. Second, it is often the case that ethics policies are not developed proactively, but rather in response to shocking events. A preferable approach would be for ecological researchers to protect their discipline from potential future scandals and the externally developed and enforced regulatory systems that often follow from them, by developing an ethics strategy for their profession.

### Benefits of an Ethics Strategy for Ecological Research

A comprehensive ethics strategy for ecological research could have at least three sets of benefits: (1) ethical, (2) empirical, and (3) political.

1. Ethical benefits: There are many ethical benefits to be gained from this effort. One benefit is the potential to resolve ethical inconsistencies in the practice of ecological research. For example, there are cases wherein two types of ecological experiments have equivalent ethical implications but are treated very differently. To illustrate, consider a hypothetical field study wherein several liters of a concentrated commercial fertilizer are introduced into a small lake to evaluate this chemical’s effect on the trout population. Because the test subject is an animal species, this study would require ethics approval from an institutional animal care and use committee. However, if the fertilizer were, instead, introduced into the lake to determine its effect on the lake’s algae, no such ethics approval would be required. Even though the trout population is affected by the study in both cases, when they are not the subjects of the research, they are not considered salient to ethics committees. This is an example of one kind of incoherence that an ethics strategy could help to minimize.
2. Empirical benefits: One of the most significant empirical benefits that can emerge from an ethics strategy for ecological research is to preserve the viability of future research projects. It can do this by raising awareness of the need to design studies that minimally impact local ecosystems. Despite the fact that ecological science seeks, in large part, to identify and measure the natural properties of ecological systems, the studies themselves inevitably disrupt these natural systems. Consequently, by directing attention to the value of minimizing the impact of research projects, the field of ecological science will preserve the integrity of future test subjects and minimize the intrusion of endogenous effects into future research programs. Consider *Example II* above: The researcher takes superlative measures to ensure the phylogenetic patterns of the local ecosystem are minimally impacted by his study; it is worth considering whether such measures should be standard practice. Overall, the development of an ethics strategy for ecological research could be integral in minimizing the impact of field studies on the natural ecosystems on which future research will depend. This, in turn, maximizes the long-term research prospects of this field.
3. Political benefits: Ecological science is a field in which the researchers themselves can speak most authoritatively and informatively on the ethically salient aspects of their discipline’s activities. The ecological researchers are experts in determining the impact of their studies on local organisms and ecosystems, and the significance of their studies in terms of knowledge production. Therefore, it is incumbent upon this discipline to self-monitor, to actively pursue ways to improve the ethical dimensions of their work, and to train future generations of researchers with the tools they need to conduct ethically well-reasoned research. Pragmatically, the implementation of a self-generated ethics strategy for ecological research would promote intra-disciplinary discussion regarding research conduct, and it could engender a positive public profile for the field both within academia and in the view of the general public and funding bodies. Additionally, the ecological research community would be signaling to external bodies its skill at transparent and ethical self-management, thereby creating a foundation from which to resist potential future efforts to impose external ethical regulations or to manipulate the research foci of ecologists.

Ensuring that ecological field studies are ethically sound requires explicit discussion of the ethics and values involved in decisions about experimental design (Bradshaw and Bekoff 2001; Minteer and Collins 2008; Schwartz et al. 2012). We recommend that ecological researchers undertake a proactive approach to developing a strategy to increase the ethical coherence of practices in their field.

## An Ethics Strategy—How?

In the preceding section, we argued for the creation of a comprehensive ethics strategy for ecological research—one that can help address the tensions and outright conflicts that currently persist within the practice of the field. In this section, we suggest a particular ethics strategy. Specifically, we propose some tools that can be useful in an ongoing process of collective ethical reflection by the community of ecological researchers, with the cooperation of applied ethicists and philosophers of biology. Critically, the approach we suggest has the resources to be responsive to the experiences and needs of ecologists, their methods, and the subject matters they study.

### Preliminary Assumptions

Our proposal is based on three preliminary assumptions that we believe to be essential for this project. Furthermore, we believe these assumptions to be both controversial in some academic circles and defensible, thus requiring explicit statement.

First: Ecological research is valuable and worth doing. This is the case even though the research by necessity involves some manipulation of natural systems. Ecologists need to collect organisms, take tissue samples, tag animals, cordon off populations, and undertake a wide variety of other tasks that are disruptive of the ecosystems that exhibit properties of scientific interest. Even purely observational studies almost invariably have some impact on the ecosystem of interest, or an adjacent one. But these intrusions can be, and typically are, justified by the information anticipated from the study.^5^ Ecological research is valuable for a wide range of reasons. Some studies, for example, can lead to better understanding of the behavioral interactions between species and their changing environments, which can very quickly translate into policies regarding land use or road design that protect the observed species. But even ecological studies that are aimed at answering foundational research questions, with apparently no immediate and practical implications, are important for developing the wider theoretical knowledge base that is needed to answer ecological questions of immediate practical import to policy and society.

Second: If a strategy is to be designed to address the ethics of ecological research, it should be developed and stewarded by the community of ecological researchers. The two most pressing reasons for this are that: (a) ecologists possess expertise crucial to the task; and (b) the strategy must be developed and maintained by the ecological community if it is to gain their support, which is crucial for its success.

Some scholars have suggested that the ethics of ecological research might best be implemented by broadening the scope of current animal care committees (Marsh and Kenchington 2004). We believe, however, that this is not feasible for ecological research. Even in the context of animal care, current committee oversight is often poorly suited to the particulars of ecological research. The animal subjects of ecological research are usually from wild populations, which can have special needs that are often better understood by the ecologist conducting the research than the veterinarian on the animal care committee, whose expertise is better suited to domesticated and laboratory animals; furthermore, the subjects are frequently members of species on which veterinarians typically have very little expertise relative to the researchers. Extending the animal care committees’ mandates to the broad range of ethical issues raised by ecological methods (which includes impacts on human communities, non-animal life, and ecosystems) increases this ‘expertise gap’ exponentially. Effective and efficient oversight of the ethical dimensions of ecological research depends critically on the skilled evaluations of ethically savvy ecologists.

Furthermore, having the strategy developed by and for ecologists is the best means of ensuring their support for and willingness to abide by these guidelines (Schwartz 2004). A survey of ecological researchers conducted at the 2013 meeting of the Canadian Society for Ecology and Evolution in Kelowna, B.C. reveals a willingness to be actively involved in the development of such an internally-created, targeted ethics strategy for the profession of ecological research. The most common objection raised at this meeting was that developing an ethics strategy for ecological research would create additional red tape and unfruitful barriers to research—that this initiative risks creating additional burdensome bureaucracy, which would be unwelcome unless it replaced and improved upon existing bureaucracy, such as the guidelines of the Canadian Council on Animal Care (CCAC).^6^ However, the best way to avoid that outcome is to have ecologists themselves at the helm because this avoids the imposition of rules by non-ecologists who may or may not understand the constraints of ecological research.

Third, a strategy designed to address the ethics of ecological research should be committed to remaining agnostic on the subject of whether non-human lives or ecosystems have intrinsic value. The notion of ‘intrinsic value’ refers to the value that something has in and of itself, separate from how it might be useful to or desired by someone. (By contrast, ‘extrinsic value’ refers to the value that something has by virtue of its usefulness or desirability to someone.) Alternatively, an ethics strategy could, at most, affirm the intrinsic value of non-human lives or ecosystems, but it should still refrain from drawing any inferences from that statement regarding the ethical legitimacy of particular practices. We follow Jay Odenbaugh’s (2003) reasoning that assigning intrinsic values to natural objects is epistemologically dubious, and that drawing moral inferences from such claims is liable to be ineffective or even harmful, given that these values cannot reliably be communicated or assumed to be shared among individuals of the same culture, let alone across cultures.^7^

This is not to say that an ethics strategy for ecological research should be entirely neutral with respect to, or free of, values. Rather, the core values should not be set by any individual ethical theory. Instead, the core values will inevitably be overlapping, sometimes in tension with one another, and created through consensus development and instrumental reasoning. This is important because the ethical reasoning of ecologists should be driven by an ongoing process of collective reflection, rather than a static document that claims to provide pat answers, or to tell how to choose between values in situations where they compete. The values that lie at the core of the ethics strategy should, rather, provide a common language—or ‘conceptual tools’—for identifying and reasoning through the salient ethical dimensions of various cases relevant to that profession. We propose a set of core values for ecological research in the next section.

### Conceptual Tool—Core Values

We propose six ‘core values’ that can provide the conceptual vocabulary for discussing the ethical aspects of ecological research. An adequate set of core values must be capable of reflecting concerns for the impact of ecological research on both human entities (such as local communities and their members, fellow ecological researchers, conservation managers, and society) and non-human entities (such as species or other taxonomic groups, populations, communities, ecosystems, and the biosphere). To this end, we suggest six principles—three that are better suited to reflect concern for human entities and three better suited for non-human entities. Collectively, these six values have the resources to address all of the ethical concerns relevant to the design and implementation of experiments in ecological research.

The first three values—freedom, fairness, and well being—are derived from Tom Beauchamp and Childress’ (1977) ‘Four Principles of Bioethics’: respect for autonomy (from which we derive freedom), respect for justice (from which we derive fairness), and respect for beneficence and non-malfeasance (from which we derive well-being).^8^ They can be used to analyze the ethical implications of ecological research with respect to human entities, such as those regarding local communities and their members, ecological researchers, and conservation managers.^9^ For instance: consideration of ‘freedom’ might inform an ecological researcher of the need to avoid jeopardizing locally valuable resources without consultation with, and consent from, local communities whose daily activities might depend on these resources (thus, the research might impinge on the freedom of these people); consideration of ‘fairness’ might inform researchers to avoid a gross imbalance in the interests of various parties effected by their research (for example, it might indicate a need to favor the interests of a less privileged group over a more empowered one); and consideration of ‘well-being’ might inform researchers regarding a ‘duty to warn’ or inform stakeholders and environmental decision-makers of their findings. While there is overlap between the applications of the values, these three are sufficiently exhaustive to permit articulation of all the ethical implications of ecological research for human entities.

The second three values—replacement, reduction, and refinement—are analogous, but not identical, to the ‘Three Rs’ of humane animal treatment, developed by Russell and Burch (1959) to guide the ethical design of research projects involving animal test subjects. In animal research ethics, ‘replacement’ refers to a preference for methods that use non-animal subjects; ‘reduction’ refers to a preference for methods that use fewer animal subjects; and ‘refinement’ refers to a preference for methods that cause less suffering and distress to animal subjects. Interestingly, ecosystem level manipulations are rarely (if ever) required to undergo any scrutiny by animal ethics committees because there are no direct animal welfare considerations, yet ecosystem level manipulations clearly have impacts on individuals and populations. These three principles related to animal research ethics will be tailored to apply to a broader range of entities than the non-human animals that are directly subjects of experimentation. Instead, it will be used to analyze the ethical implications of ecological research with respect to non-human biological entities such as individual organisms, species (or other taxonomic groups), populations, communities, ecosystems, and the biosphere. For instance: consideration of ‘replacement’ might direct ecological researchers to use simulations or natural experiments where appropriate; consideration of ‘reduction’ would guide them to minimize impacts of research on the ecosystem(s) under study; and consideration of ‘refinement’ might lead them to collaborate in order to streamline efforts.

An alternative set of core values has been proposed by ASTEC (1998) in the context of research in high-risk ecosystems. ASTEC recommends that the ‘Precautionary Principle’ should constrain all ecological research, and that within those limitations ecologists should design experiments according to four maxims: ‘movement,’ which refers to a preference for locating experiments away from sensitive ecosystems; ‘minimization,’ which refers to a preference for experiments with fewer observations where statistical significance can still be preserved; ‘modification,’ which refers to a preference for experiments that have been adapted to minimize impact on ecosystems; and ‘maximizing,’ which refers to a preference for experiments where the scientific output is as significant as possible.

Although it has been suggested that ASTEC’s conceptual system should be extended to all ecological research, we contend that our approach is preferable. While there is significant overlap between our six values and these 4Ms, ours are superior because they take into account the impact of ecological research on human entities, which is crucial to the evaluation of many ecological research projects. To illustrate, endangered species legislation in many jurisdictions can limit private property development, and backlash from local communities can limit research that ecologists can conduct. For example, a study of an endangered species of snake on Pelee Island, Ontario, was recently rejected by town council due to fears that that the discovery of the snake on private property would prevent economically important development (Jacqueline Litzgus, personal communication). Thus, the 4Ms are insufficient to evaluate all the relevant ethical implications of ecological research.

Furthermore, while the Precautionary Principle might be pertinent to research in high-risk ecosystems (although we suspect that there are considerable challenges in that domain, as well), it is not easy to apply to ecological research in general. Consider a study to assess the effects of an increasingly common environmental pollutant on an increasingly rare (though not yet officially ‘endangered’) amphibian, where the only way to assess the danger to that species involves exposing many of them to the toxin. On the one hand, the Precautionary Principle might be used to advocate that the study is impermissible because of potential harms to existing amphibians; on the other hand it might be used to advocate that the study is permissible if the study can help, in the long term, to save that species from extinction. This kind of tension between parallel and conflicting applications of the Precautionary Principle are not uncommon in ecological research, and they are bound to become more pressing as anthropogenic damage to the ecosystem escalates. Our approach would do better because it will be able to give guidance under varying conditions of uncertainty without simply, and possibly inappropriately, shifting the burden of proof to the individual ecological researcher to justify why their field study is not at all harmful.

We contend that the six core values above can provide the conceptual vocabulary necessary for an ongoing process of collective ethical reflection regarding the ethics of ecological research. In practice, they are likely to evolve over time as they are applied in consultation with ecologists to a variety of case studies. It is our anticipation that, as they are refined, a new set of values suited specifically for ecological research will emerge.

### Analytic Tool—Decision Theory

The core set of values will not be sufficient to constitute the foundation of an ethics strategy for ecological research. In addition, a procedure is needed by which guidance or advice regarding ethical field study design can be developed. Any such procedure should seek to empirically ground its analysis in the practical experiences of working ecologists. We propose a decision theoretic method that can serve as a practical means for achieving this goal.

Decision theory, broadly speaking, is the theory of human decisions through the lens of rational strategy optimization, given the presence of uncertainties, values, and (in some cases) psychological limitations. It provides an approach to decision-making that allows for the use of formal (mathematical) algorithms to derive optimal solutions (or derive proof of the absence of optimal solutions) to certain decision-making problems. We have selected the decision-theoretic approach to policy choice due to its power and effectiveness across a broad class of types of decisions.

More specifically, the decision theoretic software 1000Minds offers a ready-made, online survey program that is well suited to the task of helping ecological researchers to collectively rank a broad range of alternative scenarios stemming from value-laden decisions. 1000Minds was developed for prioritizing policy alternatives regarding healthcare (Neogi et al. 2010; Golan et al. 2011; Golan and Hansen 2012; Hansen et al. 2012), and it has been adapted to a wide variety of subjects (Ruhland 2006; Christofferson 2007; Noseworthy et al. 2009; Boyd et al. 2011; Smith and Fennessy 2011; Byrne et al. 2012). It is grounded upon multi-criteria decision analysis (also known as ‘conjoint analysis’), according to which, by presenting people with a series of decisions regarding pair-wise trade-offs, reliable predictions can be made about their relative objectives and values on particular subjects (Debreu 1960; Luce and Tukey 1964; Green and Srinivasan 1990; Green et al. 2001). This software has been used in over one hundred published studies across a variety of fields and subject matter. The algorithm at the core of this software makes use of the ‘transitivity property,’ which justifies the inference that, if a subject prefers A to B and B to C, then that subject also prefers A to C (Hansen and Ombler 2008).

To illustrate how 1000Minds could assist in assessing the ethical priorities of ecological researchers, a survey can be designed that includes the following five factors that are relevant to the design of an ethically sound field study:

1. How much does it harm non-human animals?
2. How disruptive is it of local ecosystems?
3. How disruptive is it of local human communities?
4. How likely is it that it will confound future ecological field studies?
5. How likely is it to significantly contribute to scientific knowledge?

These are not the only factors relevant to the value of a particular field study. Others include, but are not limited to, the likeliness of effectively training graduate students and the possibility that academic dishonesty (such as ‘fudging the data’) is taking place. The survey can, in principle, involve as many or few factors as necessary to obtain meaningful results.

In each stage of the survey, participants will be asked to compare two hypothetical field studies with different outcomes (low, medium, or high) on two of the six criteria. Based on the results of each selection, software will automatically fill in values for choices that can be inferred, by the principle of transitivity, from the previous selections of the person being surveyed. Thus, it is possible to obtain a complete ranking of all possible pairs of hypothetical field studies without having the surveyed person perform each ranking manually.

For example, the survey might ask an ecologist to choose which of the following two field studies is more ethical:

- Field Study A
  - Disruption to local ecosystems—Medium
  - Harm to non-human animals—Medium
- Field Study B
  - Disruption to local ecosystems—High
  - Harm to non-human animals—High

If the ecologist chooses A as the most ethical field study, the software will automatically infer that the ecologist would choose C over D in the following comparison:

- Field Study C
  - Disruption to local ecosystems—Low
  - Harm to non-human animals—Low
- Field Study D
  - Disruption to local ecosystems—High
  - Harm to non-human animals—High

Once a participant has completed a survey, 1000Minds will provide an analysis of how important she evaluates each of the five factors to be, and by how much. As more ecologists complete the survey, their evaluations can be compiled, and can even be sorted by other criteria, depending on the information requested from the surveyed ecologists (such as field of specialization or geographical location).

The main benefits of this method are that it is empirical, it is easy to implement, and it is verifiable. That is, a survey can be conducted of hundreds of ecological researchers; then, a representative sub-set of these researchers can be assembled into a focus group that vigorously debates each ranking. The results of the survey can then be compared to the results of the focus group to determine the degree of correspondence. Of additional benefit, this method can be periodically reiterated with very little cost to the individual researchers.

Furthermore, the results of the survey can be easily translated into a weighted list of priorities that ecological researchers can use as a ‘checklist’ to evaluate the ethical ‘goodness’ of particular studies. One benefit of this is that it can then help them to identify small, easy modifications that they can make to studies they propose, but which can have a significant impact on the ethical ramifications of the experiment. Additionally, it can help ecologists to efficiently make more difficult choices in the face of contentious ethical dilemmas by informing their decisions with the values collectively identified by their relevant research community.

To illustrate, we return to a hypothetical version of *Example II*: the milkweed translocation field study, where the ecologist can choose between leaving the gardens intact after the experiment or spraying the gardens with herbicide. Imagine he is relatively indifferent between the two options, but is leaning towards leaving the gardens intact to save time. On checking the ‘weighted list’ of desiderata in ethical field study design, however, he notices that choosing the herbicide option will drastically increase the ‘ethics score’ of his study; this is because the community of ecologists have collectively determined that translocation experiments that are minimally disruptive to local ecosystems contribute 5 ethics points toward the ‘ethics’ score, as opposed to only 2 points for studies that are disruptive to a medium degree, and also that studies earn 6 points if they have a negligible likelihood of confound future ecological field studies and only 1 point if this likelihood is medium. By using the herbicide, therefore, the ecologist can increase the field study’s ethics score by 8 points. Consequently, he chooses to spray the gardens with herbicide.

Similarly, we return to a hypothetical version of *Example I*: the bighorn sheep and the cougar, which is more ethically contentious. Imagine the research team is torn between trying to hunt the cougar and not; however, on checking how this decision will affect the ‘ethics score’ of the study, they notice that by hunting the cougar they significantly increase their points value on the criterion of ‘likely significant contribution to scientific knowledge,’ whereas by not hunting the cougar they do not significantly increase their points value on the criterion of ‘harm to non-human animals’ (for the sake of argument, because there is only one cougar compared with the population of sheep that will be saved). The team could, in this case, use this information to guide and ground a decision to hunt the cougar.

## An Ethics Strategy—What and Who?

### Discussion

We believe that the conceptual (six core values) and analytic (1000Minds) tools we propose together can provide the foundation for a strong, adaptive, and coherent ethics strategy for ecological research. But this is just the starting point: much work remains to be done, and many questions as yet remain unanswered.

For example: “What ethical considerations should be factored into the 1000Minds survey?” and “Who is considered a member of the ecological research community?”. These two questions are interrelated insofar as we contend that the community itself must decide what factors are most relevant to it; thus, as different sub-communities of ecological researchers are identified by common traits—such as by their areas of specialization or by the countries in which they conduct their experiments—different factors of responsible research design will be relevant to varying degrees. The data collected during a 1000Minds survey can be subdivided by field of study to permit more precise identification of priorities more relevant to specific subject of study (trees, turtles, freshwater ecosystems, etc.), investigative approach (phylogenetics, trophic relationships, behavioural ecology, etc.), or research institution type (university, government, non-governmental organization, industry, etc.). As such, it makes sense to cast the net widely, sending the survey to many researchers and sorting the results afterwards to determine salient categories and priority differences.

Importantly, we are approaching these questions from an open perspective. We believe that relevant sub-communities will emerge through iterative feedback on the developing ethics strategy. Our working assumption is that the best way to approach this sub-division of ecological researchers is to identify the sub-groups into which they have aggregated by way of professional societies. Based on what we have observed from these communities, we expect that what will be most effective is for the national organization representing ecological researchers (such as the CSEE) to hold the responsibility for developing and maintaining the ethics strategy for its country, and that it will contain specific clauses relevant to matters uniquely pertinent to particular subfields or regions. But this is speculative. The important point is that the ethics strategy must be an dynamic and flexible document if it is to be effective; we believe it is a strength of our proposal, rather than a weakness, that it is adaptive in this respect.

## Conclusion

We have argued that the ecological research community needs to develop a targeted ethics strategy to guide research design. It is essential that ecological researchers themselves develop this strategy if it is to be accurate and relevant in its recommendations as well as successful in its uptake. These researchers (in consultation with bioethicists and philosophers of biology) are best suited to identify ethically relevant practices, and they should do so before a method is imposed by an external body—possibly with more invasive and less effective results.

We have suggested a set of six core values—freedom, fairness, well being, replacement, reduction, refinement—that have the conceptual resources to provide vocabulary for discussing the ethically salient dimensions of ecological research studies. Furthermore, we suggest an analytic method for empirically evaluating the priorities of ecological researchers with respect to the ethically responsible design of field experiments. 1000Minds provides a means for identifying how ecologists prioritize various ethically salient factors in research design. The results of these online surveys can be aggregated, analyze, and used to develop guidance for ecological researchers.

The development of an ethics strategy for ecological research will have at least three sets of benefits: ethical, epistemic, and political. Additionally, it can help to structure graduate student training in the ethics of ecological research. Currently, ecology graduate students tend to view ethics as a hurdle to overcome (that they need to ‘get the ethics’ to be able to run their experiments) rather than as a tool for enhancing research practices and improving the field. This assistance could be a great boon, therefore, to the professors tasked with assisting these students to develop critical reasoning skills regarding the ethics of their work.

Importantly, an ethics strategy must be adaptive to the evolving needs of the ecological research community, and of particular sub-communities of researchers. It could, for example, assist ecologists in mitigating the pressures of partisan politics on research design. This issue is increasingly salient in Canada and the USA, where claims of political interference in ecological research have recently been in the headlines (Goldenberg 2012; Shen 2013); it might be less relevant, however, to ecological researchers in other countries, such as Norway or New Zealand. By contrast, New Zealand and Canadian ecologists might share a particular interest in respecting the traditions, rights, and well being of indigenous communities—a concern that might not be shared to such an extent by researchers in Norway or the USA.

We propose a particular ethics strategy—one that hinges on an ongoing process of collective ethical reflection that the community of ecological researchers, with the cooperation of applied ethicists and philosophers of biology, can use to address the needs we have identified. This approach is important due, in part, to the escalating urgency of pollution, climate change, and other destructive anthropogenic phenomena that are monitored, directly or indirectly, by ecological researchers.

## Footnotes

1 Unless otherwise indicated, quotations are taken from a survey of ecological researchers undertaken by the authors at the 2013 meeting of the Canadian Society for Ecology and Evolution. Identities of those quoted have been withheld in accordance with an agreement of confidentiality. .

2 For example, researchers have documented the importance of this in the context of the managed relocation of species such as polar bears in response to climate change (Minteer and Collins 2008; Schwartz et al. 2012).

3 Environmental ethics is distinct from the ethics of ecological research insofar as the former deals with broader questions concerning the relationships between humans and the rest of nature, including, for example, the ethical implications of policy options regarding land use, consumption, green energy sources, extinction, and duties to future generations. The ethics of ecological research, by contrast, is focused on the experimental design options of the field of ecology.

4 This merits further discussion from an epistemological perspective, because ‘uncertainty’ comes in many forms, with very different implications.

5 If this seems uncontroversial, consider that the subject discussed here overlaps with the literature on environmental ethics wherein there is a school of thought that advocates protecting the sanctity of wild ecosystems in their untouched state, even if this means humans will be unable to study those systems (Turner 1994).

6 The CCAC is the national organization responsible for maintaining research ethics standards with respect to non-human animals.

7 Note that a commitment to agnosticism regarding the intrinsic value of ecosystems and organisms in no way contradicts a commitment to the value of foundational or ‘basic’ research on these entities. Just as a mineral or an element might not have intrinsic value in and of itself, yet conducting basic research on it might be valuable insofar as it extends human knowledge and opens further avenues of research, some of which might even have practical or technological benefits, basic ecological research remains valuable regardless of whether the entities they research are accorded intrinsic moral value.

8 ‘Ethical principlism’ has been central to bioethics since its inception in the 1970s (CIHR et al. 2010), when it was introduced as a method for analyzing ethical issues (in medical ethics, originally, but later in a larger array of bioethical sub-fields) and arriving at broad consensus in bioethical debates by drawing on values that are shared by a wide range of people despite their other philosophical disagreements. For example, a Kantian deontologist will disagree with a Utilitarian consequentialist on matters of ethical theory, but by focusing on shared ethical principles, they can be expected to reach agreements on many practical issues that are significant for making real-life decisions (Davis 1995). On this approach, mid-level principles such as ‘respect for autonomy’ and ‘respect for justice’ are specified without being theoretically ranked. Although we adopt some of the conceptual apparatus of this principlist approach, we do not subscribe to the more radical view that these principles reveal a common morality shared by all rational humans. Rather, we offer these as a heuristic device to facilitate deliberations regarding ethical issues relevant to this particular field.

9 This is not to suggest that all stakeholders would need to be actively consulted prior to any ecological field research, but rather that their interests should be taken into account when designing field studies. Often these interests can be inferred by researchers, who interact with the relevant groups periodically. The extent to which these interests should be taken into account, and the identification of cases wherein active consultation is required, would be determined by the researchers and their peers through discussion and debate.
